# In General Practice
**Dr. Grey,* by Stephen Andrew. London: Greening and Co. Price 6s. net.


**Published:** 1911-04-15

**Authors:** 


### SPECIAL ARTICLES.

### IN GENERAL PRACTICE.*

The doctor in fiction is a very variedly coloured specimen of professional humanity, who has not always been drawn with sympathy and with true artistic feeling. The fine pictures that Trollope gave us, the no less fine—though in a different sense—sketches of Molière, Samarow, and Crabbe, are too well known to need comment, though it is possible that Mr. Maarten Maartens' caricature of the enthusiastic bacteriologist with his solicitous "Mind!" to a nervous wife, may not be so familiar to our readers. On the whole the novelists have treated the doctor well. He has been overdrawn it is true, and the picture, even when the artist has had the best and most pious intentions, has been crudely coloured, whether it was limned with the gentle hand of a Jerrold or harshly brushed in with the vigour of a Turgénieff. The gross exceptions—a recent example, presumably compounded from colonial elements with which we confess we are imperfectly acquainted—are travesties which are too unreal to be taken seriously, and whose only justification, if they have any justification at all, is the fact that they may, on rare occasions, point a political moral. Of this nature is Defoe's catalogue of Whig physicians, enumerated with all the biting satire so often degenerating into scurrility which the author of "The Character of a Whig" possessed. But the several component parts, in no matter what class we examine, of the doctor in fiction are, it must be admitted, very badly fitted together and with one or two rare exceptions (witness Dr. Thorne) the pictures drawn by lay writers are grotesquely unconvincing. No medical novelist, of the stamp of Weir Mitchell, has as yet given us his conception of his colleagues as types in fiction; the few doctors who have written novels ranking among the curiosities of literature have been woefully unfortunate in sketching their own class.

### A General Practitioner's Book.

"Dr. Grey" appears to us an exception to this general rule. Mr. Stephen Andrew, whose nom-de-plume hides the name of a well-known medical practitioner in the Birmingham district, is thoroughly familiar with the doctor's life, and his sketch, slight as it is, and showing traces as it does very frequently of amateur handling, is one that appeals because it is on the whole very truthful, depending for its command of interest not on extraneous embellishments but on the central figure itself. We wish we had the space to review Mr. Andrew's book with the fulness that its importance demands. We have read it with interest, and while the author would perhaps be the first to agree with us that it is not a great book, nor even a book that is likely, through the slightness of plot and the comparative unattractiveness of the characters, to prove popular, we are convinced that it is a very important landmark in the literary career of its writer. The man who can write with such simplicity, who can describe ordinary scenes with such sympathetic ease, who shows that he has observed commonplace things and commonplace men with keen attention, and who, moreover, impresses his readers with the sense that he has thought and not merely imagined and sketched, is a man who needs careful watching by the critics. Great writers do not flaunt across the literary firmament like meteors trailing in the wind: they rise like specks in the Milky Way, and gradually creep from asteroidal to planetary splendour. For our part, the reading of "Dr. Grey" has made us interested in its author's literary future; we await his next work with eager anticipation, and look forward to even larger results in the future.

### Hospital Life.

For "Dr. Grey" is a book that well repays reading, and though it necessarily, since it deals largely with medical questions, appeals more strongly to us of the profession, it should receive attention, no less, from the mass of novel readers. Its story is very simple. Briefly put, it is the unvarnished record of a newly qualified medical man who ventures into the depths and shoals of private practice. The first few chapters introduce us to Arthur Grey, the hero, a conjoint diplomate just finishing his term at "St. Dorothea's Hospital"—an institution the identity of which is almost painfully obvious. Mr. Andrew's thumbnail sketches of some of the hospital staff show that he possesses humour.

### Locum Tenens Work.

The hospital is left behind and Grey begins his real life. From this point everyone who has had the slightest experience of private practice will agree with us that Mr. Andrew handles his hero admirably, and it is this part of the book, to which the lighter introductory chapters are fitting preludes, that claims more serious consideration. The manner in which Grey acquits himself of his assistantship, how he buys a practice after going the round of a dozen, and how he fares in his new home—all this Mr. Andrew must be permitted to tell for himself and it would be unfair to him and his readers alike to reveal how the story ends.

### The Problem of Poverty.

To a mind, like Lamb's, so "painfully introspective" as Mr. Andrew's, the elemental problems of the day call insistently for treatment. We do not think the author is acquainted with the poetical philosophy that is enshrined in the "Ideas," of Eduard Douwes Dekker, but there are passages in his book—bits in Grey's conversation especially, —which strongly suggest the table talk of the good doctor who attempted to cultivate the fertile field of Walter Pietresen's mind. "Poverty, vice, and philanthropy," remarked the doctor, "or what so

* *Dr. Grey*, by Stephen Andrew. London: Greening and Co. Price 6s. net.

often passes for the last, are three varieties of the same disease which co-ordinately cause, support, control, and complete each other." Fourier, almost a hundred years before, expressed the essentials of the problem of poverty in slightly different phrasing, and in Mr. George Cope's fine sonnet is found the germ of the sociological dogma that Mr. Andrew's journalist, O'Brien, inculcates into Grey:—

Ye have them always with you, then—these poor?
A cumbrous load—some reason a reproach—
. . . . . . . . . . .
Nay, by the splendour of God! have they not you?
Galls not your goad the lacerated side?
Mars not your curb the mutilated tongue?
What frauds but yours the living walls have dried
And sown despair their barren souls among?

Enthusiastically, yet with moderation and restraint that are as rare as they are welcome in a popular exposition of the faults of our existing systems of medical and poor relief, the case for reform is presented to the reader. And no popular representation of the salient facts rivals in clearness, in tersity of presentation, in detail of instance, this simply written story of a general practitioner's struggles in a Birmingham back slum. Here we are shown the manner in which the clubber is dealt with—the degradation attendant on parish relief, the utter misery of poverty in tenement dwellings. Inevitably the question arises "How are we going to mend it?" That it is mendable, nobody who has studied the matter denies. That there are very diverse views on the subject of amendment the clash of the several reports of the Poor Law Commissioners prove. We have no intention of discussing the subject here. Mr. Andrew obviously inclines towards the opinions of the Minority report, and his story is in itself a very powerful argument in favour of the suggestions made in that report. Here, in fine, we have the thoughts of a general practitioner on one of the most salient questions of the day. As such the book has a high practical value, especially when we bear in mind that the writer is intimately acquainted with the things he writes about and that he has taken a more than *dilettante* interest in the subject of poverty and poor relief. At the same time "Dr. Grey" is an admirable presentation of the case for the general practitioner. It is, as we have already said, a record of general practice, very faithfully given, with few side issues brought in that are irrelevant to the major discussion. Every page calls for quotation, but we refrain, for no excerpt however full can do justice to the whole. The book must be read in its entirety, and we sincerely trust that practitioners will buy it, will see to it that a copy is placed on the shelves of the village library, and will call attention to it in every possible way, by discussing it with laymen who take a serious interest in these problems. It is a book that will well repay careful reading. The apparent slightness of the narrative is no drawback to the manner in which the case is presented, for the book is not merely a concatenation of controversial statements: it is a well-digested summary of facts given in clear and simple language. And the central figure, that of Dr. Grey, painted with sympathetic delicacy and with a charming simplicity that compels admiration for the sketch as a whole, deserves to rank as one of the truest and most telling portraits of a medical man in fiction.

